# Auditing the clinical usage of deep-learning based organ-at-risk auto-segmentation in radiotherapy^☆^

**DOI:** 10.1016/j.phro.2025.100716

**Published:** 2025-01-30

**Authors:** Josh Mason, Jack Doherty, Sarah Robinson, Meagan de la Bastide, Jack Miskell, Ruth McLauchlan

**Affiliations:** Department of Radiobiology and Radiation Physics, Imperial College Healthcare NHS Trust, Charing Cross Hospital, Fulham Palace Road W6 8RF London, UK

**Keywords:** Clinical audit, Deep learning auto-segmentation

## Abstract

For 18 months following clinical introduction of deep-learning auto-segmentation (DLAS), an audit of organ at risk (OAR) contour editing was performed, including 1255 patients from a single institution and the majority of tumour sites. Mean surface-Dice similarity coefficient increased from 0.87 to 0.97, the number of unedited OARs increased from 21.5 % to 40 %. The audit identified changes in editing corresponding to vendor model changes, adaption of local contouring practice and reduced editing in areas of no clinical significance. The audit allowed assessment of the level and frequency of editing and identification of outlier cases.

## Highlights

• Deep learning auto-segmentation editing was audited for 1255 patients.
• A reduction in organ at risk editing was observed after an initial period.
• Audit data identifies outlier cases and trends in organ at risk editing.

## 1 Introduction

Deep learning auto-segmentation (DLAS) models are widely used for delineation of organs at risk (OARs) for radiotherapy planning [1]. Typically DLAS is evaluated in a limited number of patient cases and by a small number of users before clinical implementation. Risks of DLAS in clinical practice include failure to handle outlier cases, automation bias [2], user complacency and DLAS model updates. To address these risks, audits of DLAS editing should be performed, however this loses some of the time saving gained from DLAS, it is not feasible to audit every patient manually and monitoring ongoing clinical usage in a busy department with large numbers of independent users is challenging.

There are few studies investigating usage of DLAS in the clinic: an audit of clinical editing of head and neck (H&N) OARs by Nealon et al [3] found a difference in editing practice for one dosimetrist, and decreased editing over time. Vaasen et al [4] audited more than 700 breast and lung patients focused mainly on spatial location of edits and found changes such as different computed tomography (CT) scanning protocol impacted the level of adjustments required. Brouwer et al [5] found variation between users and highlighted spatial locations that required frequent editing in analysis of 12 months of H&N OAR editing. Analysis of differences between DLAS and clinical contours can help select clinically relevant cases for model re-training [6] and assess consistency in clinical trial quality assurance [7].

We investigated the value of automated script based auditing to analyse trends in clinical editing of DLAS contours. The audit was intended for monitoring usage of DLAS in the post-evaluation clinical phase.

## 2 Material and methods

MVisionAI GBS^TM^ v1.2.3-v1.2.5 (MVisionAI, Helsinki, Finland) OAR contouring was used for 1336 patients over a period of 18 months. The anatomical models used (and number of patients) were: male pelvis (495), breast (244), female pelvis (190), abdomen/thorax (240), H&N (162), whole body (5). All OAR contours were manually reviewed and adjusted for clinical treatment planning.

Approval was obtained through local clinical governance procedure for clinical audit. A script compared the DLAS contour to the final approved contour for each OAR in each patient, by calculating the surface Dice similarity coefficient (sDSC) [8] (3 mm tolerance). The script also calculated the number of times an OAR contour was used unedited (geometrically identical). The script was run at 2 month intervals giving 9 consecutive time periods with 100–200 patients in each period. In 6 % of patients the script failed due to not being able to select the correct case leaving 1255 patients included in the audit. Due to limited DLAS licenses, alternative DLAS software was used for simple breast patients, brain patients, and for simple prostate patients in time periods 4–9. These patients were not included in this audit.

Approximately 40 users performed OAR contouring including clinical oncologists and dosimetry/physics staff with minimal staff changes during the audit period. MVision guidelines matched our clinical protocols for breast [9], brachial plexus, spinal cord, parotid [10], oesophagus, liver, kidney, vessels [11], brainstem, globe, lens, optic chiasm, optic nerve [12], bladder, femoral head [13], rectum [14], small and large bowel [11], [13], lung, spinal canal [15], bronchus, chestwall, trachea [16], heart [15], [16].The analysis only included clinically relevant OARs with a dose constraint defined in the clinical protocol. For example, for a simple prostate treatment only bladder, rectum and femoral heads were included in the audit although the DLAS male pelvis model included many additional OARs.

Some changes were made during the evaluation period: local practice for femoral head contouring was changed after the initial 2 month period to include the femoral neck, matching the DLAS model; after ∼ 4 months the DLAS software was configured to grow the spinal cord by 1 mm as it was felt the DLAS spinal cord was less generous than standard local practice; and the DLAS OAR for heart including superior aspect of pulmonary artery (hereafter referred to as heart + a pulm), as defined in UK SABR consortium guidance [16], was initially under-contoured superiorly but improved following feedback to the vendor and a model update ∼ 6 months post-implementation.

## 3 Results

**Fig. 1:**
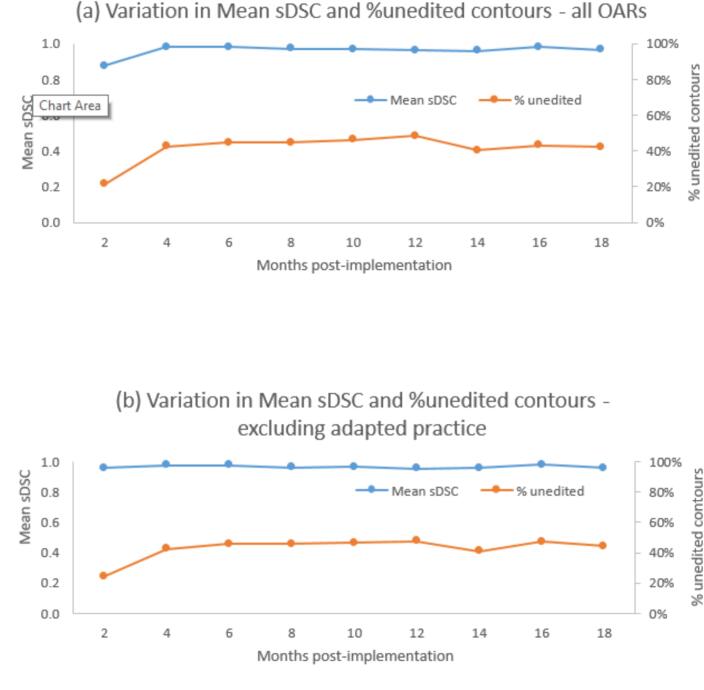

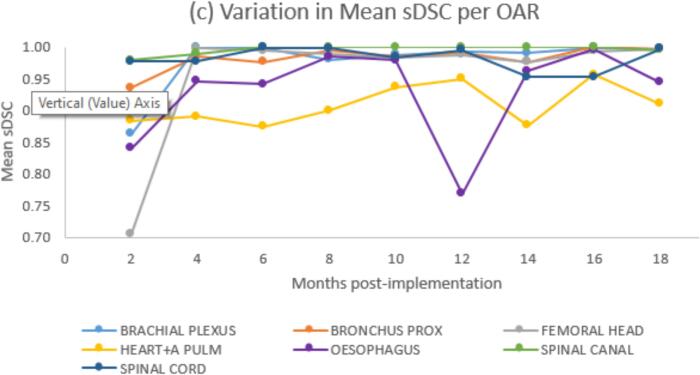
(a) Variation by month of audit of Mean sDSC and percentage of unedited contours for all OARs combined. (b) Variation by month of audit of Mean sDSC and percentage of unedited contours excluding femoral head, heart + a pulm and spinal cord, for which local practice was adapted after initial 2–4 months. (c) Variation in Mean sDSC by month of audit for selected OARs.

Averaged across all OARs, mean (standard deviation, range) sDSC increased from 0.87 (0.12, 0.71–1) to 0.97 (0.05, 0.82–1) during the audit and the percentage of unedited contours (range for different OARs) increased from 21.5 % (0 %-73 %) to 40 % (0 %-90 %) during the audit (Fig. 1a). Excluding OARs for which changes were made during the audit (femoral head, heart + a pulm and spinal cord as explained above) mean sDSC was similar across all time periods, but percentage of unedited contours increased from 24.5 % to 40–47 % (Fig. 1b).

For most OARs the mean sDSC did not vary with time, OARs that did show variations are as follows (Fig. 1c): Brachial plexus showed reduction in editing attributed to expanding use of brachial plexus contours from SABR only to breast/lung planning where the OAR has less tight tolerances. Bronchus prox showed a reduction in editing which could reflect automation bias although patient numbers are small so this could be random variation. Reductions in editing of femoral heads, spinal cord and heart + a pulm reflect the changes made for these OARs during the audit, as explained above. For oesophagus occasionally only the inferior section is contoured by the DLAS, and this happened for 5/11 patients during time period 6. Spinal canal reduction in editing was due to DLAS outlining the whole cord and users becoming more used to not editing regions far away from the target.

Mean and standard deviation sDSC values (Table 1) were used to assess the level and frequency of editing and to highlight cases where the amount of editing was outside the usual range. Examples of outlier cases identified by the audit included the following: patient with a 2.5 cm^3^ bladder, patient with transplanted kidneys, oesophagus cases where the DLAS didn’t produce a good contour (this relates to the issue described above), parotid contour impacted by CT artefact, rectum contours with unusual anatomy or unusual filling, unusual trachea anatomy − split into three sections at the carina, a brain patient where the incorrect DLAS tool had been used resulting in a poor quality brainstem DLAS contour, bronchus prox and heart + a pulm contours where the DLAS contour was incorrectly edited. In all these cases the DLAS contour had been manually corrected, or the issue had no clinical impact.

**Table 1:** Percentage of ROIs unedited and mean and standard deviation (SD) of surface Dice similarity coefficient (sDSC) across all time periods.

	**Total ROIs**	**% ROIs unedited**	**Mean sDSC**	**SD** **sDSC**		**Total ROIs**	**% ROIs unedited**	**Mean sDSC**	**SD** **sDSC**
**Bladder**	563	15 %	0.98	0.04	**Lens**	76	71 %	1.00	0.01
**Brachial Plexus**	58	43 %	0.98	0.05	**Liver**	45	31 %	0.98	0.05
**Brainstem**	113	19 %	0.96	0.12	**Lung**	671	62 %	1.00	0.01
**Breast**	171	63 %	0.91	0.19	**Oesophagus**	150	39 %	0.93	0.18
**Bronchus Prox**	27	4 %	0.97	0.02	**Optic Chiasm**	24	25 %	0.93	0.10
**Chestwall**	29	31 %	1.00	0.00	**Optic Nerve**	46	41 %	0.92	0.18
**Femoral Head**	1156	36 %	0.95	0.13	**Parotid**	201	42 %	0.98	0.05
**Globe**	64	44 %	1.00	0.00	**Rectum**	523	10 %	0.96	0.08
**Heart**	327	34 %	0.97	0.04	**Small Bowel**	127	38 %	0.87	0.24
Heart + A Pulm*	31	10 %	0.90	0.05	**Spinal Canal**	301	83 %	0.99	0.03
**Humeral Head**	191	73 %	0.99	0.03	**Spinal Cord**	231	55 %	0.97	0.08
**Kidney**	259	69 %	0.98	0.08	**Trachea**	228	59 %	0.97	0.06
**Large Bowel**	94	51 %	0.93	0.16	**Vessels**	12	17 %	0.92	0.06

* Heart including superior aspect of pulmonary artery, as defined in UK SABR consortium guidelines [9].

User feedback was that following the initial period, editing reduced due to a combination of (i) increased standardization in local practice due to the DLAS contours following guidelines closely (ii) users reducing the number of adjustments made in spatial locations that will have no impact on the treatment plan.

## 4 Discussion

The amount of editing of contours decreased after the initial ∼ 2 month period of usage reaching a steady state with ∼ 40 % of contours requiring no editing. The audit identified variations in editing due to vendor model changes, adaptation of local contouring practice and reduced editing of areas with no clinical significance. The audit allowed assessment of the level and frequency of editing of OARs and identification of outlier cases. For femoral heads, spinal cord and heart + a pulm a reduction in editing over time was expected due to changes in local practice or model updates., However if these OARs are excluded from the analysis the trend of reduced editing still remains.

There are few studies in the literature evaluating clinical usage of DLAS software or tracking variations in usage over time. An audit by Langmack et al [17] found benefits in terms of clinical workflows and staff work-life balance in a post-implementation audit, but did not look at variation of quantitative metrics over time. Our audit did not detect substantial change in practice beyond the initial period, whereas the audit by Nealon et al [3] suggested individual dosimetrists were affected by automation bias – the difference may be because only minor editing of DLAS contours was required in our study.

According to user feedback, reduction in editing is due to increased standardisation of practice and adapting to only making edits that have potential clinical impact. We do not know whether this adaptation is automation bias or a conscious decision to change practice, it is likely a combination of both. Automation bias may not be a problem if the DLAS matches guidelines well, but could be harmful if the DLAS consistently fails to contour accurately. Strategies to mitigate automation bias could include giving regular training to staff to highlight the potential issue and show examples from the audit, or to periodically require contours to be performed manually. Another potential issue with adoption of DLAS is training new staff. In our department we continue to require staff to learn to contour manually before using DLAS.

The combination of percentage of structures that are unedited and mean sDSC values gave insights into level of OAR editing. For example OARs such as bladder, brainstem and rectum consistently needed editing, but only by a small amount. OARs such as kidneys, lungs and spinal canal often needed no editing, but sometimes were edited a small amount. Outlier sDSC values were identified and investigated, in one case (oesophagus) this resulted in an issue with the DLAS software being identified and fed back to the vendor.

The audit used sDSC which has been shown to be more closely correlated to editing time than DSC [8]. Vaasen et al [18] also showed that sDSC was more clinically relevant and correlated to time saving than DSC or Hausdorff distance, also finding that active path length was even better, unfortunately this couldn’t be used in the audit due to the scripting framework used. Automated auditing can assist busy clinical departments to conform with the ESTRO/AAPM recommendations [19] for QA and upgrade testing of DLAS.

For the majority of OARs, there was no variation in mean sDSC across the time period of the audit. However for some OARs there was variation over time and in most cases this reflected the impact of change to local practice or updates to vendor models. In some cases though the change may reflect clinicians gradually and possibly sub-consciously changing their practice and being guided by the DLAS.

The audit method had the following limitations: 3 mm tolerance for sDSC may not be the most appropriate value for all OARs; for serial OARs such as spinal cord the sDSC was impacted by variations in adjustment of the superior-inferior extent even though this often has no clinical significance; any OARs where the clinician has deviated from standard naming conventions were missed by the script (standardised structure templates are used to minimise these cases but the script cannot count how many times OARs are missed); where an OAR was edited less than usual, it may just be because the clinician felt the edits were not clinically relevant (an example was breast which is sometimes used as a target and sometimes as an OAR); average values in the data presented will be dominated by the OARs that were used most frequently. Individual outlier cases and some OARs having small numbers of occurrences in each time period caused some variation in the results, however we would rather have an outlier flag unnecessarily than miss a change in DLAS output or user behaviour. Additionally it would be useful to audit results for individual users separately and to evaluate the dosimetric impact of changes in level of editing, which are difficult to implement with our current systems but are areas for future development of the audit process.

In conclusion the audit method provided a method to track changes in user behaviour and the impact of updates to DLAS vendor models in a clinical setting of a large radiotherapy department, provided a reasonable level of assurance and quality control of DLAS editing, and allowed individual cases of interest to be identified and investigated.

## CRediT authorship contribution statement

**Josh Mason:** Conceptualization, Methodology, Software, Formal analysis, Supervision, Funding acquisition. **Jack Doherty:** Software. **Sarah Robinson:** Formal analysis. **Meagan de la Bastide:** Formal analysis. **Jack Miskell:** Formal analysis. **Ruth McLauchlan:** Supervision.

## Declaration of competing interest

The authors declare that they have no known competing financial interests or personal relationships that could have appeared to influence the work reported in this paper.
